# Relationship of Extrinsic Risk Factors to Lower Extremity Injury in Collegiate Ballet Dancers

**DOI:** 10.3389/fbioe.2022.878448

**Published:** 2022-05-11

**Authors:** Pi-Yin Huang, Chia-Wei Lin, Amornthep Jankaew, Cheng-Feng Lin

**Affiliations:** ^1^ Department of Physical Therapy, College of Medicine, National Cheng Kung University, Tainan, Taiwan; ^2^ Institute of Allied Health Sciences, College of Medicine, National Cheng Kung University, Tainan, Taiwan; ^3^ Physical Therapy Center, National Cheng Kung University Hospital, Tainan, Taiwan

**Keywords:** pointé shoes, training, musculoskeletal injuries, ballet, lower extremity

## Abstract

Ballet dancers are thought to be at higher risk of lower extremity injury. This objective of this study was to describe the self-reported incidence, location, and factors associated with lower extremity injury in collegiate ballet dancers. Two hundred and forty-nine female ballet dancers responded to a questionnaire that addressed their injury event/location, dance behavior over the past 2 years, and overall dance history. Behaviors assessed included the following: types and number of shoes worn (pointé shoes/ballet slippers), wear time, training time (session frequency and duration), use of warm-up/cool-down, and use of a strengthening program and lower extremity accessory. Overall dance history included age of the onset of training, total years of experience, and number of dance styles. Backward multivariable logistic regression analysis was used to determine the extent to which variables measured were associated with injury. Ankle injury was the most prevalent injury. Years of wearing pointé shoes (adjusted odds ratio = 1.21, *p* = 0.01) and days/weeks in pointé shoes (adjusted odds ratio = 1.26, *p* = 0.04) were associated with an increased risk of injury; while additional strengthening (adjusted odds ratio = 0.39, *p* = 0.02) and use of lower extremity accessories during classes/rehearsals (adjusted odds ratio = 0.64, *p* = 0.01) were protective associations. These findings suggested that the use of pointé shoes, lower extremity accessories, and additional exercise should specifically be recorded during evaluation of injured ballet dancers; and must be considered potential factors to modify during rehabilitation.

## Introduction

Ballet is an art exercise that requires long-term training and has high physical performance demands. Ballet dancers are at a high risk of injuries, especially those considered overuse or cumulative injuries (Costa et al., 2016; Smith et al., 2016; Fuller et al., 2019; and Fuller et al., 2020). Most ballet dancers are exposed to strenuous training at an early age (Twitchett et al., 2009), before their musculoskeletal system matures. This has been reported to have a negative effect on skeletal sites (Amorim et al., 2017).

Gender may affect the risk of injury since female ballet dancers have to dance on tiptoes more frequently than male ballet dancers, and female dancers dance in pointé shoes which place their bodies under unique lower extremity stresses. Consequently, female ballet dancers sustain a higher incidence of foot and ankle injuries (Kadel, 2014; Vosseller et al., 2019). A pointé shoe is a type of footwear commonly worn by ballerinas when performing pointé work that allows weight bearing on tiptoes with ankle plantarflexion called en pointé. The basic construction of a pointé shoe is a stiffened toebox with a platform, a stiff shank to support the foot arch while en pointé, and enclosed with a fabric cover. This can place increased biomechanical stress on the lower extremities (Aquino et al., 2021).

Both intrinsic and extrinsic factors have been suggested as potential risk factors for ballet injury. Studies have suggested that ballet injuries are associated with intrinsic factors such as female gender, age, reduced functional turnout, pronated foot, insufficient ankle plantar flexion range of motion, and previous contralateral inversion ankle sprain (Kenny et al., 2016; Campbell et al., 2019). The extrinsic risk factors among female dancers proposed to increase ballet injury were training errors (Kaufmann et al., 2021), inappropriate training methods (Kaufmann et al., 2021), technique demands and style of dance (Yin et al., 2019), shoes, environmental temperature (Fong Yan et al., 2011), floors and costumes (Barringer and Schlesinger, 2004), and psychosocial factors (Byhring and Bø, 2002). Although ballet shoes are an important consideration, their role is rarely discussed in the current literature. In addition, long-term exposure to ballet training may predispose dancers to injury. The experience or the years of ballet training has been shown as an associated factor of a number of painful sites (Nunes et al., 2002) but not a predictor of injury in ballet (Hiller et al., 2008). As previous studies have not yet considered some other extrinsic factors such as ballet shoes and shoe paddings, it was, thus, necessary to examine the possible risk factors of ballet injury to further prevent ballet injuries.

As the dancers may not seek advice or treatment from medical professionals when the injury occurs, the incidence from an injury register may be underestimated. A questionnaire is a self-report research method often used to establish injury prevalence and incidence (Steffen et al., 2008; Harrison et al., 2020) and may better reflect the injuries in dancers rather than injuries recorded in health service systems (Moita et al., 2019). In addition, the questionnaire screening method can increase self-awareness of their limitations in dancing and determine the relationship between physical characteristics (e.g. strength or range of motion) and injury risks through the tracking of injury history information (Steinberg et al., 2012). Therefore, there is a rationale to use self-report to establish injury rates in ballet dancers (Ekegren et al., 2014; Caine et al., 2016). The purposes of this study were to describe the following in ballet dancers 1) self-reported lower extremity injury incidence and location and 2) factors associated with lower extremity injury.

## Materials and Method

### Participants

Female collegiate ballet students in dance schools (*N* = 249) were invited to this retrospective study and filled out the study questionnaire. In the current study, female collegiate ballet students were of our interest because pre-professional dancers had inferior performance skills and tended to practice for more hours than professional dancers. The inclusion criteria were collegiate ballet students with regular ballet training. Exclusion criteria involved professional dancers or ballet students with a history of lower limb surgery. This study was approved by the Ethical Committee of the Institutional Review Board (IRB) of the National Cheng Kung University Hospital, and all the participants gave informed consent before participating in this study.

### Questionnaire Design and Content

A questionnaire of ballet injury was designed based on the literature (Byhring and Bø, 2002; Nunes et al., 2002) and feedback provided by ballet instructors. The questionnaire was pre-tested with three dancers to clarify ambiguous questions or response options. All the questions used to establish incidence used the previous 2 years as the referential time period. This questionnaire included five sections that addressed demographics, dancing history, ballet shoes, training history, and injury history. Demographic information included date of birth and medical conditions. The dancing history section addressed the age when the dancer began ballet training, years of ballet training (ballet experience), and current dance styles. The training history section included ballet training frequency (days/weeks), ballet training duration per session (minutes), ballet training duration per day (minutes), warm-up time and cool-down time (minutes), frequency of training in pointé shoes (days/weeks), and additional strengthening programs other than dancing routines. The section addressing shoes included the duration of wearing ballet slippers (time changes to new ballet slippers), number of years wearing pointé shoes, use of lower extremity accessories during classes/rehearsals (ballet training classes, ballet technique classes, and rehearsals but not on stage), and the number of ballet slippers and pointé shoes. The section addressing use of lower extremity accessories included flexible ankle sleeves, ankle taping, forefoot taping, toe taping, forefoot pad, toe pad, and toe separator. The injury history section acquired information on the number of injuries, injury site, how the injury occurred, impact of the injury on training, activities of daily life (ADL), and whether long-term discomfort was observed after injury. The ADL questions inquired about walking, stair ascent and descent, and indoor ambulation. Long-term discomfort was defined as pain, soreness, and any other discomfort that is caused by the injury and if symptoms persisted for at least 3 months.

### Injury

Injury was defined as a physical insult to the lower extremity, limiting a dancer from regular training routine or dancing routine for at least 24 h. Anterior and posterior body charts were included in the questionnaire for dancers to mark the injury site. A description of the injury event was also recommended when reporting the injuries. The principal investigator was present to explain each item and answer questions raised by dancers who participated in the present study. The participants were encouraged to recall their injury status and situations.

### Data Analysis

Mean and standard deviation of the independent variables were calculated. The distribution of lower extremity injures was determined and reported as a percentage of total injuries. The number of dancers affected by the injury in their ADL and in long-term discomfort was determined as the percentage of total dancers.

### Statistical Analysis

Backward multivariable logistic regression was conducted to evaluate how strongly a given dancing variable was associated with the injury compared to other dancing variables, and a likelihood-ratio test was used to determine the significance of those associations. The potential risk factors were items from the dancing history, training history, and ballet shoe sections, while the occurrence of lower extremity injuries was determined as a dependent variable. All the statistical analyses were conducted through SPSS version 15.0 (SPSS Inc. Chicago, United States). A *p* value less than 0.05 was used as an indication of statistical significance.

## Results

A total of 249 questionnaires were distributed but only 239 copies (96% response rate) were completed and used for final study sample analyses. The mean age of the 239 subjects was 20.64 (2.17) years. The achieved power ranged from 0.56 to 0.85 with 239 subjects, and this was calculated based on determined odds ratio, R-square, and mean and standard deviation of factors. A high lower extremity injury incidence (86.6%) was reported with a total of 207 injured ballet dancers in the past 2 years. The average total number of injuries in the past 2 years was 2.67 (2.10), and the average injury site was 2.05 (1.76). Dancers sustained the greatest number of injuries to the ankle (34.5%) (Table 1). This was followed by the knee (27.7%), and foot (combined forefoot and rearfoot = 12.7%).

**TABLE 1:** Distribution of injury to the bilateral lower extremities sustained over the past 2 years.

Injury sites and effects	Number of Injuries	Percentage^a^
Mid- or fore-foot	48	8.7
Rear foot	22	4.0
Ankle	191	34.5
Lower leg	23	4.2
Knee	153	27.7
Thigh/hip	61	11.0
R1 Groin	55	9.9
Total	553	100

a Number of injuries of each part relative to total number of injuries.

Mean and standard deviation of the independent variables are summarized (Table 2). A total of 116 out of 239 (48.5%) ballet dancers reported that the injury affected their ADL that included walking, stair ascent and descent, and indoor ambulation. Sixty-nine of the 239 (28.9%) ballet dancers reported that an injury resulted in discomfort that bothered them for at least 3 months.

**TABLE 2:** Mean (SD) and percentage of independent variables.

	Ballet dancers (*N* = 239)
Number of years wearing pointé shoes	2.5 (2.4)
Ballet slipper–wearing duration (month) (time changes to new ballet slippers)	20.6 (28.3)
Number of ballet slippers	2.2 (2.6)
Number of pointé shoes	1.3 (1.0)
Ballet training frequency (days/weeks)^a^	4.0 (1.5)
Ballet training duration per session (min.)^a^	111.8 (36.4)
Ballet training duration per day (min)^a^	225.6 (35.4)
Training in pointé shoes (days/weeks)	1.7 (0.9)
Warm-up time (min.)	18.2 (10.8)
Cool-down time (min.)	8.8 (8.0)
Age at which the dancer begins to dance ballet (year)	10.6 (3.0)
Ballet dancer experience (year)	8.5 (2.7)
Number of dance types	3.2 (1.0)
Use of lower extremity accessories during classes/rehearsals	65.3%
Additional strengthening	51.5%

a Ballet training in addition to performance and rehearsal.

The model demonstrated high predictive ability, with 86% of the dancers classified correctly as injured using the predictor variables. Time in pointé shoes including the number of years of wearing (adjusted odds ratio = 1.21; *p* = 0.01) and days/weeks in pointé shoes (adjusted odds ratio = 1.26; *p* = 0.04) was associated with a higher likelihood of injury. Conversely, the use of lower extremity accessories during classes/rehearsals (adjusted odds ratio = 0.64; *p* = 0.01) and additional strengthening (adjusted odds ratio = 0.39; *p* = 0.02) were significant protective factors (Table 3). These adjusted odds ratio represented that on average, a 1 year increase in years of wearing pointé shoes resulted in a 21% increased likelihood of lower extremity injuries; a 1 day/week increase in wearing pointé shoes resulted in 26% increased likelihood of injury; for those using lower extremity accessories, there was a 36% decreased likelihood of injury, and for those participating in additional strengthening, a 61% decreased likelihood of injury.

**TABLE 3:** Significant predictors to lower extremity injury in ballet dancers.

	*B*	*p*-value	Adjusted odds ratio	95% CI for the adjusted odds ratio
Year of wearing pointé shoes	0.187	0.013*	1.21	0.93–1.56
Use of lower extremity accessories during classes/rehearsals	−0.447	0.010*	0.64	0.24–1.69
Days/weeks in pointé shoes	0.234	0.041*	1.26	0.73–2.20
Additional strengthening	−0.952	0.017*	0.39	0.15–1.03

Statistical method: backward multivariable logistic regression.

*p < 0.05.

## Discussion

This study revealed high rates of overall and activity-limiting lower extremity injuries in a large sample of collegiate ballet dancers. In addition, the study determined that the number of years and days/weeks wearing pointé shoes was associated with an increased injury risk, while a strengthening program and use of lower extremity accessories during classes and rehearsals were protective factors.

We obtained a high response rate (96%), similar to the 99% (98 out of 99) reported previously by Askling et al., and higher than that reported in another previous study (80%; 41 out of 51) (Askling et al., 2002; Byhring and Bø, 2002). Thus, the findings of this study may provide a confident estimate of the actual incidence of self-reported lower extremity injuries in ballet dancers.

### Distribution of Injuries

Our study agreed with previous findings that ankle injury was the most common injury in ballet dancers, followed by knee and the foot injuries (Kadel, 2014; Vosseller et al., 2019). Conversely, Garrick et al. (Garrick and Requa, 1993) reported that the highest prevalence of ballet injury was in the foot (23.9%), then the lumbar spine (23%), and followed by the ankle (13.3%). The difference in the prevalence of foot and ankle injuries in the studies by Garrick et al. and ours may come from differences in the target population (professional vs. collegiate dancers), definition of injury (medical/insurance cost vs. dance-related injury), and investigation period (3 vs. 2 years). Caine et al. (Caine et al., 2016) reported that the three common injury locations were the hip (17.54%), followed by the knee (14.91%) and ankle (14.91%), and the tibia and foot (8.77%). The differences between Caine et al. and our study might come from the differences in ballet levels (pre-professional vs. collegiate dancers) and gender (mixed vs. female). Our questionnaire focused only on lower extremity injuries because upper extremity injuries were relatively uncommon (4%) (Caine et al., 2016). In addition, the reason why professional ballet dancers have more injuries in the hip/lumbar area may come from the dancing choreography as they often have more solos and movements with leg splitting or extreme lumbar extension with large lordosis (Mira et al., 2019).

In addition, we examined the incidence of injury to the foot by forefoot and rearfoot segments to further understand the distribution and possible injury mechanism of foot injuries. Common injuries to the forefoot include sesamoid disorders, metatarsal fractures, and flexor hallucis longus dysfunction (Kadel, 2014; Vosseller et al., 2019). Sesamoid plays an important role for increasing the mechanical advantage of flexor hallucis brevis and for bearing weight while walking and rolling over the foot onto demi-pointé or full pointé. Thus, sesamoid injuries might cause prolonged disability. One of the most common stress fractures in dancers is located on the base of the second metatarsal bone due to repetitive loading in a demi-pointé posture (Kiel and Kaiser, 2022). In addition, large pressure is sustained at the first and second metatarsal bones during grand jeté, predisposing the second metatarsal bone to stress fractures. Dancers with flexor hallucis longus dysfunction have pain while jumping and full pointé because the flexor hallucis longus tendon passes through the tarsal tunnel and is easily entrapped at several sites (Kiel and Kaiser, 2022). Although we did not specify the types of foot injuries in ballet dancers, the higher injury incidence of forefoot injuries (8.7%) than rearfoot (4.0%) may indicate that repetitive weight-bearing on tiptoes and ball of the foot predisposes the forefoot to injury.

### Influences of Injury

Almost all dancers reported an injury in the previous 2 years; however, the severity of these was unknown. We inquired whether the injury affected dancer ADL and resulted in long-term discomfort. This might indicate that injuries were not mild. The aspects of ADL that were most affected by the injury were walking and stair ascent/descent which are of relatively low intensity but frequently executed compared with dancing movements. Dancers with long-term discomfort might exhibit compensatory movement patterns and that may contribute to the likelihood of further injury. It is recommended that dancers receive adequate care promptly after injury for better recovery and for prevention of long-term discomfort.

### Ballet Shoes and Foot Accessories

Two main types of ballet shoes: soft ballet slippers and pointé shoes are often worn during training or on stage. Different aspects of footwear properties such as hardness and morphology might affect loading rate, balance control, and shock absorption (Luftglass et al., 2021), and these factors might be associated with injuries. However, very few studies investigated how ballet shoes influence lower extremity injuries. A study of Cunningham et al. (1998) indicated that the mechanical properties of the pointé shoe toe box varied among shoe types. They also reported that the first three criteria for selecting a pair of pointé shoes for ballet dancers were fit, comfort, and box shape; however, they did not present how forces were attenuated by the toe box (Cunningham et al., 1998). The entire shank (sole) of the pointé shoe is rigid. A stiff toe box encloses the forefoot and a flexible cloth makes up the remainder of the pointé shoes. In contrast, ballet slippers are made of canvas or leather and are very flexible. Ribbons are for an esthetic look and are also used to stabilize the ankle inside the pointé shoes but the stabilization effect is minimal. Wakes and Caudwell (Wakes and Caudwell, 2010) further suggested that many ballet injuries were caused by lack of support and shock-absorption provided by technique shoes and an inadequate fit. From this aspect, ballet shoes, particularly pointé shoes, may need to be refined with appropriate shock absorption material or padding to prevent injury. Pointé shoes using manmade plastezote-type materials with an extra layer of cushion built in the toe box such as Gaynor Minden and Bloch which are now on the market. However, its effect on preventing injury deserves future investigation.

We found that use of lower extremity accessories during classes and rehearsals was a protective factor, while the number of years wearing pointé shoes was the risk factor significantly associated with injury occurrence. A previous investigation (Aquino et al., 2021) showed that the number of painful sites experienced per dancer was larger in the pointé group than that in the non-pointé group. This result along with our finding suggests that the longer years of wearing pointé shoes, the longer the duration of the feet being exposed to a restricted space with poor shock absorption. Not surprisingly, this would predispose dancers’ lower extremities to musculoskeletal injuries as repetitive jump-landing and heel rising are quite common during ballet dance.

Ballet dancers can choose foot and ankle accessories that might reduce forces on their feet. The lower extremity accessories dancers used in the present study includes flexible ankle sleeves, ankle taping, forefoot taping, toe taping, forefoot pad, toe pad, and toe separator. Based on the results of our retrieved questionnaires, dancers who used lower extremity accessories during classes/rehearsals did have a lower total number of injuries than those who did not. McPherson and his colleagues confirmed that ballet technique shoes with padding can redistribute pressure and reduce total force with the most efficient one being padding with foam pads to metatarsals, toes, and a high arch support (McPherson et al., 2019). While our study cannot confidently attribute the cause and effect, it provides weak evidence that these strategies did have an impact. Use of taping and bracing to prevent ankle injuries is common in many sports. Previous investigations in other sporting activities showed that injury rates are lower if lower extremity accessories are used (Hamlyn et al., 2012; Muñoz-Barrenechea et al., 2019). Based on the findings of emerging evidence to date, it is suggested that the use of lower extremity accessories during classes and rehearsals may be suggested for ballet dancers.

Dancers selected their shoes based on the fitting and shape, not the duration or strength of the shoes (Barringer and Schlesinger, 2004). Milan (1994) suggested that toe abscesses would occur if the pointé shoe was too small, and excessive movement of the heel would result if the shoe was too large. Thus, additional shoe space is required to avoid toe squeezing and to prevent secondary injuries such as mal-alignment. Bickle (*Bickle et al., 2018*) also reported that the deterioration in the pointé shoe structure with excessive wear may increase the risk of foot and ankle injuries due to a malalignment of foot and pressure caused on joints. Selection of the proper accessory or equipment for preventing pointé-related injuries is important. For example, the toe caps and callous padding would counterwork to the impact of structural limitation and foot injury (Barringer and Schlesinger, 2004). In addition, it is generally accepted that ballet dancers do not use flexible ankle sleeves or ankle taping on stage for esthetic sake. Therefore, development of new external accessories that can function well with an acceptable esthetic look should be considered. Biomechanical and material studies may inform how to optimally design these devices.

### Training History

The routine training profile (days/weeks in pointé shoes and additional strengthening) is another strong protective factor to injury in the current study. Almost all the dancers performed warm-up and stretching exercise, but only 51.5% (123 out of 239) of them received an additional muscle strengthening program. The proportions of those who received additional strengthening exercises were similar to 45% reported by Byhring and Bø (2002). The flexibility program is deemed an important component in ballet training. Nevertheless, according to the length and tension relationship, excessive muscle length would be detrimental to force production (Bohm et al., 2021), thus adversely affecting performance and leading to an injury. Our findings again showed that dancers who participated in an additional strengthening program were less likely to incur an injury. This finding was partially supported by Koutedakis and colleagues (Koutedakis and Sharp, 2004) who reported that hamstring and quadriceps muscle strengthening is beneficial to professional ballerinas because of the increased thigh muscle torque and also the prevention of muscle torque decrements after dance routines. This suggested an important message for dance educators and dancers that muscle strengthening is an essential component for prevention of injury, and they should know how to implement it into their training routine effectively (Premelč et al., 2019). Unfortunately, the self-report design of this study did not allow us to define what kinds of strengthening exercises were, or should be, performed by ballet dancers. Physical therapists need to define optimal training programs for ballet dancers. Prospective studies are needed to determine if well-designed strengthening programs are beneficial for ballet dancers in terms of performance and injury prevention. Frequency of ballet training (days/weeks) and hours of ballet training per day, warm-up time, and cool-down time were not significantly associated with injury in this study. These findings were inconsistent with the results of a previous study where professional ballet dancers who practiced more than 5 hours per day were at a higher risk of lower limb stress fracture than those dancing less than 5 hours per day (Kadel et al., 1992). Others found that injury mechanisms and types of injuries correlated with dancing techniques and practice time (Costa et al., 2016). In the current study, the total hours of dancing per day (including other types of dancing) were not recorded, and no participants practiced ballet more than 5 hours per day on average (mean = 226 min, Table 2), except for rehearsal. The training duration per day presented here excluded the time for rehearsal and performance because rehearsal and performance are subject to season and schedule (Kadel, 2014; Jeffries et al., 2020), and it would be difficult to record training duration during special seasons in a retrospective study. In addition, college dancers were dancing much less than professionals. All these may explain why training duration and frequency had no significant relationship with injury in our study. Furthermore, there could be a relationship between time spent in pointé shoes and total time dancing (co-linearity), which might have meant that the stronger predictor is the time spent in pointé shoes. Other studies may not have separately measured time of dancing and time in pointé shoes. Finally, the type of injury measured was different across these studies and may explain the discrepancy between findings. It has been generally accepted that adequate rest is necessary to avoid fatigue that can contribute to sports injuries. Others suggested that poorly organized ballet classes or rehearsals, especially with long breaks between dancing activities, may result in cooling of the soft tissues (Ramel and Moritz, 1994), and may also lead to injury.

### Dancing History

We expected that the age of starting ballet training might be a predictor because strenuous ballet training received at an earlier age may have a negative effect on the peak bone mass accumulation and bone mass density (Amorim et al., 2021). A study (Munoz et al., 2004) showed that the menarche delay of young female ballet dancers was positively correlated with years of dance before puberty. Although our study did not find a significant relationship, we may not have a sufficient range of age onset (5–14 years old) to detect this effect. We also did not focus on bone quality/injury or menstrual disorders. So, the effects of age onset may not be as important for lower extremity injury. Previous findings suggested that strenuous training received at an earlier age for young athletes would influence bone development and biomechanical properties of the bone and that might put young athletes at the risk of overuse injuries (Munoz et al., 2004; Amorim et al., 2021). Future studies on the association between young ballet dancers or the age starting to dance ballet and injury rate are necessary. In addition, other related intrinsic risk factors such as joint range of motion, muscle strength, and body structure should also be considered concomitantly with the study of extrinsic risk factors due to the fact that intrinsic risk factors may be a determinant to extrinsic factors and have the association with lower limb injuries among recreational dancers (Steinberg et al., 2012).

The number of years of ballet dancing did not contribute significantly to the injury. This finding is similar to that of Hiller et al. who presented that the years of ballet training was not a predictor of lateral ankle sprain in ballet,^8^ but this result is inconsistent with that of a previous investigation which showed that the years of ballet dancing could predict the number of painful sites in recreational young dancers (Nunes et al., 2002). The inconsistent results may be due to different criteria of recruited participants. Nunes et al. (2002) recruited only recreational dancers who danced in pointé shoes or not while we recruited collegiate ballet students. The inconsistent level of ballet training may contribute to a different presentation of injury or discomfort. A clear operational definition or classification of the experience level should be established to eliminate the discrepancy in future study.

In our study, ballet dancers on average received about three other types of dance training, such as contemporary (26.8%), traditional folk dance (27.5%), and tap (17.7%), but this was not associated with injury. It is possible that different dance styles have different risk profiles. For example, modern dance requires kneeling and barefoot, while ballet requires deep knee flexion in grand-plié or bearing weight on tiptoes in pointé shoes. However, Bronner et al. (2003) suggested that fewer muscular imbalances might be caused when multiple dance styles are used. However, there is little evidence to the overall impact of dance style. Only one study mentioned that some ballet dancers attended other types of dance training such as tap, jazz, and modern dance, but they did not analyze the association between this factor with the number of injuries or injury rate (Nunes et al., 2002). Future studies should focus on how a combination of different types of dance or cross-training affects the injury.

Our data indicated that each lower extremity part underwent multiple injuries even though the mechanism of injury or type of injury may be different. Repeated injury to the same location may indicate inadequate management of the original injury or early return to dancing with incomplete recovery (Rauh et al., 2007). Recurrent injury tends to be a major problem for dancers (Rinonapoli et al., 2020). A previous study reported that about 93% of professional dancers were affected by persistent or recurrent injuries (Negus et al., 2005). These findings support the need for lower extremity injury prevention programs in ballet dancers.

### Study Limitations

Some limitations of this study need to be acknowledged here. Most importantly, our study relied on retrospective recall, and this leaves it open to recall bias. Despite this potential bias, self-report may better reflect the injuries in dancers rather than injuries recorded in health service systems (Moita et al., 2019). Jenkins et al. (2002) indicated that recall periods of greater than 2 months may induce a bias and are possible to underestimate the injury. The ability to remember exposures we measured as potential risk factors may have been more subject to recall bias. This would have tended to attenuate the strength of our associations and may have missed important associations. In addition, we were unable to control the intrinsic risk factors such as functional turnout or pronated foot by use of a retrospective questionnaire. Other related intrinsic risk factors such as joint range of motion, muscle strength, and body structure should be considered concomitantly with extrinsic risk factors, given the fact that the intrinsic risk factors may be a determinant to the extrinsic factors and have association with lower limb injuries among recreational dancers (Steinberg et al., 2012). The findings are not representative for professional ballerinas as the questionnaires were answered only by collegiate ballet students. However, the collegiate ballet students are important to clinical practice because collegiate ballet students had lower performance skills and tended to practice more hours than professional dancers. In addition, they may manifest highly incorrect techniques showing a positive correlation with lower limb injury risks (Steinberg et al., 2012). Furthermore, the survey study methodology did not allow us to explore the nature of reported injuries or exposures in detail. Thus, we are unable to define specific elements of shoe designs/devices, training schedules, strengthening exercises, or other aspects of risk prevention programs. The inherent reliability and validity of the questionnaire were not determined, though this questionnaire was designed based on the literature and dance instructors and pre-tested with three dancers before being formally launched. Furthermore, the definitions of injury and risk factors vary across studies. Standardization of these across future studies might enhance the comparability of results. Future studies are needed and ideally should be longitudinal cohort studies with accepted definitions of type, location, and severity of injury. There should be some validation of self-reported injury by a physical examination at least a subset of the respondents/injuries. Dancers with different ballet levels should also be included to investigate how the experience, training program, and choreography may affect injury. Again, standardized definitions of these across studies would enhance comparability.

## Conclusion

This study suggests that lower extremity problems are very prevalent in ballet dancers and that greater time wearing and training in pointé shoes may increase risk. Conversely, the use of lower extremity accessories during classes and rehearsals and additional strengthening exercises may decrease the risk of lower extremity injury. There is a need for prevention and treatment programs for ballet dancers and these factors may be considered in developing those interventions.

## Data Availability

The original contributions presented in the study are included in the article/Supplementary Material, further inquiries can be directed to the corresponding author.
